# Soybean Antigen Protein-Induced Intestinal Barrier Damage by Trigging Endoplasmic Reticulum Stress and Disordering Gut Microbiota in Weaned Piglets

**DOI:** 10.3390/molecules28186500

**Published:** 2023-09-07

**Authors:** Lei Wang, Yujia Liu, Daoliang Zhang, Hongyan Ding, Shibin Feng, Chang Zhao, Jinjie Wu, Xichun Wang

**Affiliations:** 1 College of Animal Science and Technology, Anhui Agricultural University, Hefei 230061, China; 2 Anhui Provincial Key Laboratory of Livestock and Poultry Product Safety Engineering, Institute of Animal Husbandry and Veterinary Medicine, Medicine Academy of Agricultural Sciences, Hefei 230061, China

**Keywords:** endoplasmic reticulum stress, soybean glycinin, *β*-conglycinin, autophagy, gut microbiota

## Abstract

Endoplasmic reticulum (ER) stress is a crucial factor in the pathogenesis of intestinal diseases. Soybean antigenic proteins (*β*-conglycinin and soy glycinin) induce hypersensitivity reactions and intestinal barrier damage. However, whether this damage is associated with ER stress, autophagy, and the gut microbiome is largely unclear. Therefore, in this study, we aimed to investigate the effect of dietary supplementation with soy glycinin (11S glycinin) and *β*-conglycinin (7S glycinin) on intestinal ER stress, autophagy, and flora in weaned piglets. Thirty healthy 21-day-old weaned “Duroc × Long White × Yorkshire” piglets were randomly divided into three groups and fed a basic, 7S-supplemented, or 11S-supplemented diet for one week. The results indicated that 7S/11S glycinin disrupted growth performance, damaged intestinal barrier integrity, and impaired goblet cell function in piglets (*p* < 0.05). Moreover, 7S/11S glycinin induced ER stress and blocked autophagic flux in the jejunum (*p* < 0.05) and increased the relative abundance of pathogenic flora (*p* < 0.01) and decreased that of beneficial flora (*p* < 0.05). In conclusion, 7S/11S glycinin induces intestinal ER stress, autophagic flux blockage, microbiota imbalance, and intestinal barrier damage in piglets.

## 1. Introduction

Endoplasm reticulum (ER) is an important eukaryotic organelle that participates in many cellular processes, including intracellular processing and synthesis of proteins, storage of calcium ions, and metabolism of lipids and carbohydrates [1]. Stress factors induce accumulation of unfolded and/or misfolded proteins and cytosolic calcium imbalance in the ER, leading to unfolded protein response (UPR) and ER stress [1]. The intestinal epithelium constitutes a key barrier and messenger between the lumen environment and the host immune system and is particularly vulnerable to ER stress [2]. Microbial toxins and metabolites may disrupt the intestine environment and lead to ER stress in intestinal epithelial cells [3,4]. Moreover, epithelial cells with high secretory capacity, such as goblet cells and Paneth cells, constantly produce many large proteins with complex structures, which may be potential targets of protein misfolding and UPR activation [4]. Notably, ER stress in the intestinal epithelium causes a series of adverse cellular reactions, including redox imbalance, impaired autophagy flux, uncontrolled inflammatory reactions, and apoptosis, all of which are involved in the pathogenesis of intestinal barrier dysfunction [5].

The diverse bacteria living in the gut constitute the gut microbiome, and there are approximately 10 trillion species. A dynamic and relatively proportional balance between various microorganisms must be maintained in the gut for correct functioning of the intestinal barrier [6]. McKenzie et al. identified the “nutrition–gut flora–physiology axis” as an important link between diet, gut flora, and allergic diseases [7]. Gut flora has co-evolved in a specific host ecological niche for thousands of years and continues to adapt during symbiosis to maintain homeostasis with the host immune system, thus playing a key role in the host immune response. When the composition of the gut microbiome changes, its function may also change, which in turn affects the hosts’ health [8]. This condition is known as gut microbiota disorder and can lead to respiratory, digestive, and immune system diseases [9].

Soybeans are rich in essential amino acids and are an important source of plant-based proteins for humans and livestock. However, various antinutritional factors in soybean, such as trypsin inhibitors, soybean agglutinin, soybean antigen protein, and tannin, can seriously affect the health of animals as well as the digestion and absorption of nutrients [10]. Among them, *β*-conglycinin (7S glycinin) and soy glycinin (11S glycinin), which account for approximately 70–80% of the total protein content of soybeans, are two of the most immunologically active multi-subunit globulins. Both 7S glycinin and 11S glycinin are thermally stable, acid- and base-resistant, and resistant to enzymatic digestion, and their antigenic activity is difficult to destroy during product processing. Due to the special physiological growth performance of piglets, the digestive organs are not yet developed and functional, and the digestive enzyme activity is low. Weaned piglets fed a diet containing soy antigenic protein showed symptoms of digestive disorders, diarrhea, reduced production performance, and even death, resulting in huge economic losses to pig farms [11,12]. Our team have revealed that 7S glycinin and 11S glycinin damaged the intestinal barrier via the nuclear factor κB/mitogen-activated protein kinase signaling pathway and destroyed mitochondrial function [13,14]. However, whether the damage of 7S glycinin and 11S glycinin on the healthy intestinal barrier is associated with ER stress, autophagy and the gut microbiome is largely unclear.

In this study, we fed 7S glycinin and 11S glycinin to 21-day-old weaned piglets to establish an animal model and assessed the effects of 7S glycinin and 11S glycinin on the intestinal epithelial barrier and intestinal flora. In addition, the effects of 7S glycinin and 11S glycinin on intestinal epithelial ER stress and autophagic flux were investigated. Our results may provide valuable insights into the mechanism of intestinal barrier dysfunction caused by 7S glycinin and 11S glycinin, thus providing a theoretical basis for attenuating their sensitizing effects.

## 2. Results and Discussion

### 2.1. 7S/11S Induced Growth Performance Loss and Allergy in Weaned Piglets 

**Figure 1:**
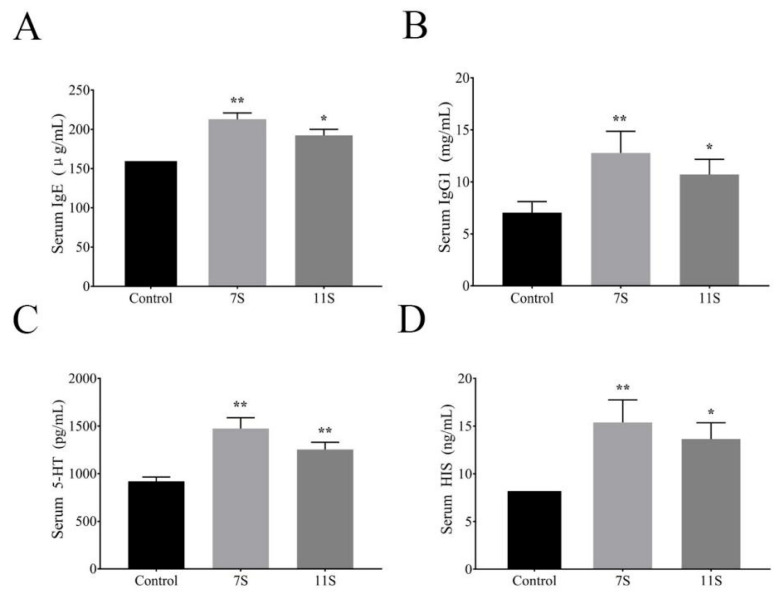
(**A**) IgE level of weaned piglets in each group. (**B**) IgG1 level of weaned piglets in each group. (**C**) 5-HT level of weaned piglets in each group. (**D**) HIS level of weaned piglets in each group. Data are shown as mean ± standard deviation (SD). * *p* < 0.05, ** *p* < 0.01, compared with the control group.

The supplementation of 7S and 11S significantly decreased the ADG of piglets, but significantly increased the F/G (*p* < 0.01; Table 1). As shown in Figure 1, the levels of IgG1, IgE, HIS, and 5-HT in the serum of weaned piglets in groups 7S glycinin and 11S glycinin were significantly higher than those in the control group. Meanwhile, all piglets in the 7S glycinin and 11S glycinin groups showed watery diarrhea symptoms, whereas piglets in the control group showed no diarrhea symptoms. The diagnosis of food allergy is usually easy if there are sudden signs of oral swelling, urticaria or pruritus after feeding, but it is rarely recognized that allergy may also be the culprit of diarrhea [15]. These results suggest that 7S- and 11S-induced diarrhea in piglets may be associated with allergic reactions.

### 2.2. 7S/11S Altered the Cecal Microbiota Diversity and Composition of Weaned Piglets

**Figure 2:**
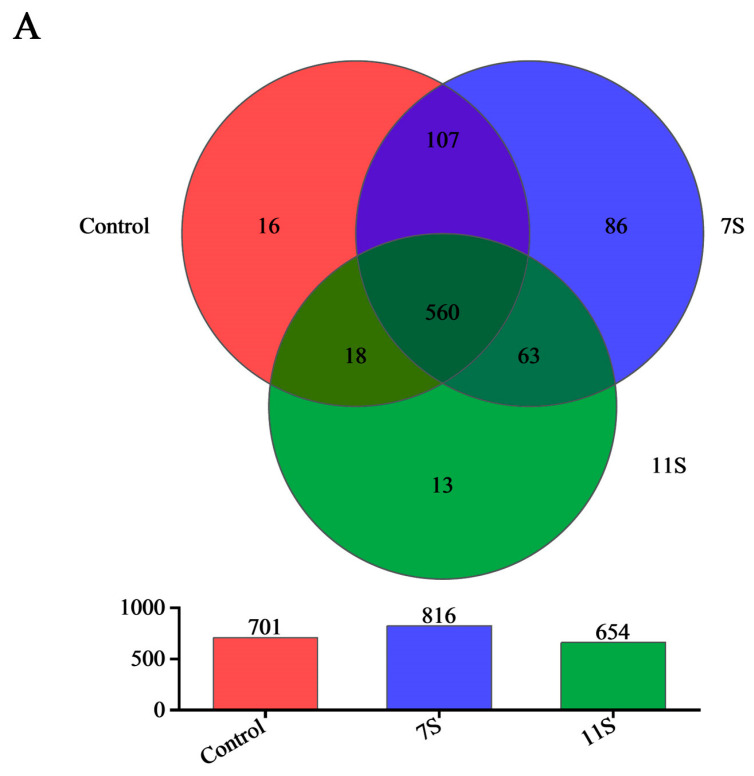

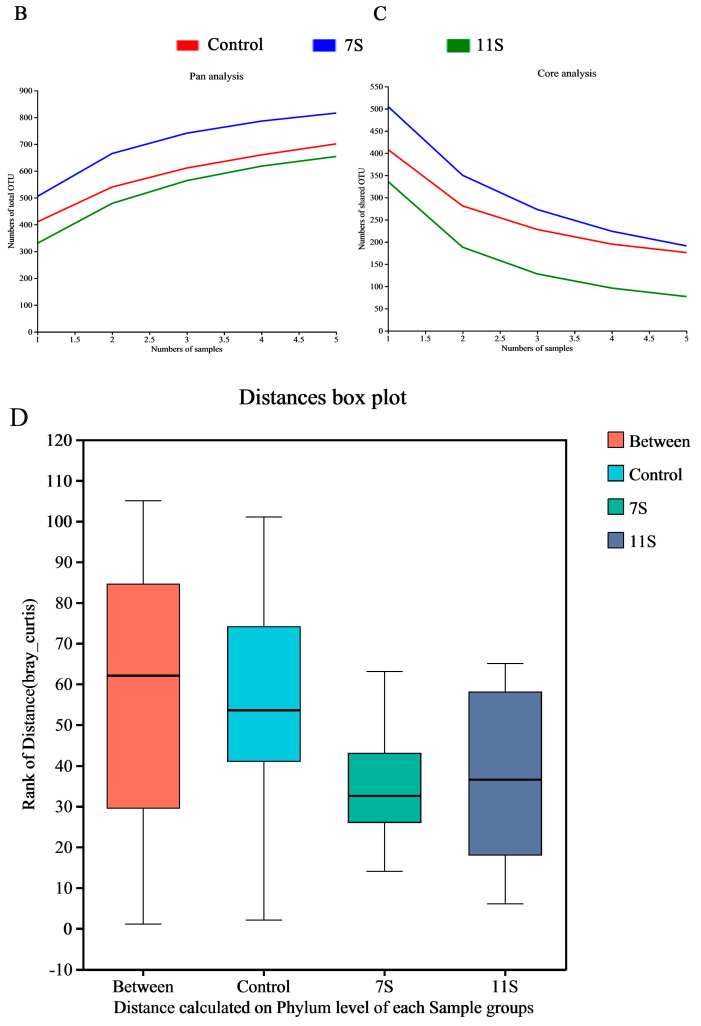
(**A**) Venn diagram. (**B**) Core species analysis. (**C**) Pan species analysis. (**D**) ANOSIM analysis.

It has been widely demonstrated that disorders of the intestinal flora are closely associated with the development of allergic diseases [16]. HiSeq sequencing analysis of 16S rRNA in 15 weaned piglets was performed to investigate the effects of 7S glycinin and 11S on cecal bacterial communities. As shown in Figure 2A–C, with the increase in intestinal contents of weaned piglets in each group, Core OTU and pan OTU tended to be flat, suggesting that the sample size is sufficiently representative for this sequencing. Results of ANOSIM analysis are displayed in Figure 2D, *R* = 0.3013 (0 < *R* < 1), *p* = 0.012 (*p* < 0.05), which show that the differences between groups were greater than the differences within groups, indicating that the grouping in this trial was scientifically significant. 

**Figure 3:**
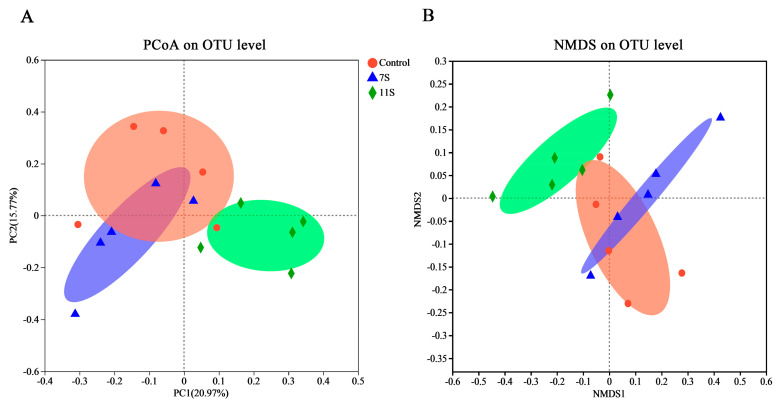
(**A**) PCoA analysis. (**B**) NMDS analysis.

As shown in Table 2, compared with the control group, 11S decreased the Chao1, Ace, Shannon, and Simpson indices and richness of cecal microbiota (*p* < 0.05). Interestingly, compared with the control group, the Shannon, Simpson, ACE, and Chao indices and richness in the 7S glycinin group showed a change in trend, but the difference was not statistically significant. Principal component analysis (PCoA, Figure 3A) and non-metric multi-dimensional scaling analysis (NMDS, Figure 3B) of intestinal contents showed that the microbial groups of control and 7S group piglets partially overlapped. However, 11S piglets were distinguished from microbial groups observed in control and 7S piglets, indicating the differences between the sensitized group and the control group. 

**Figure 4:**
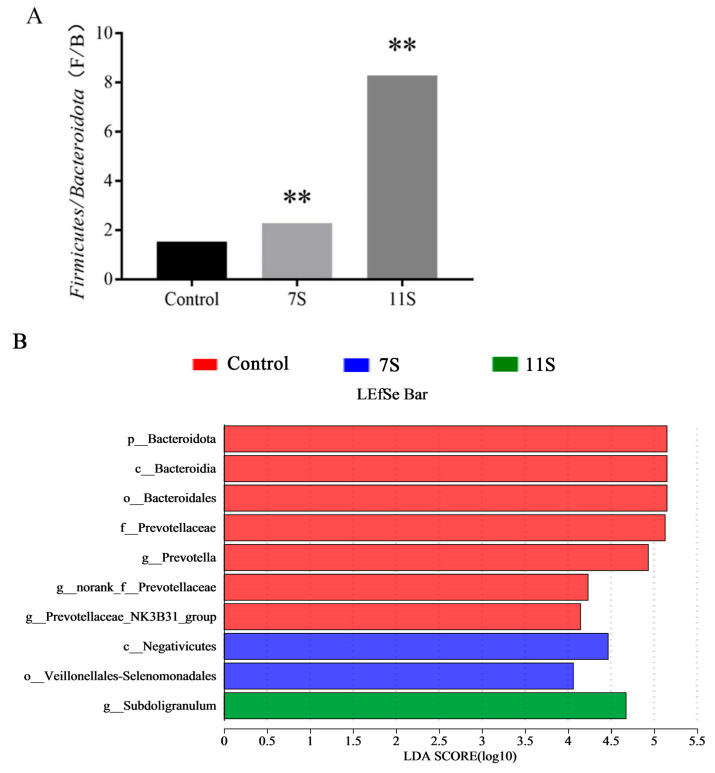

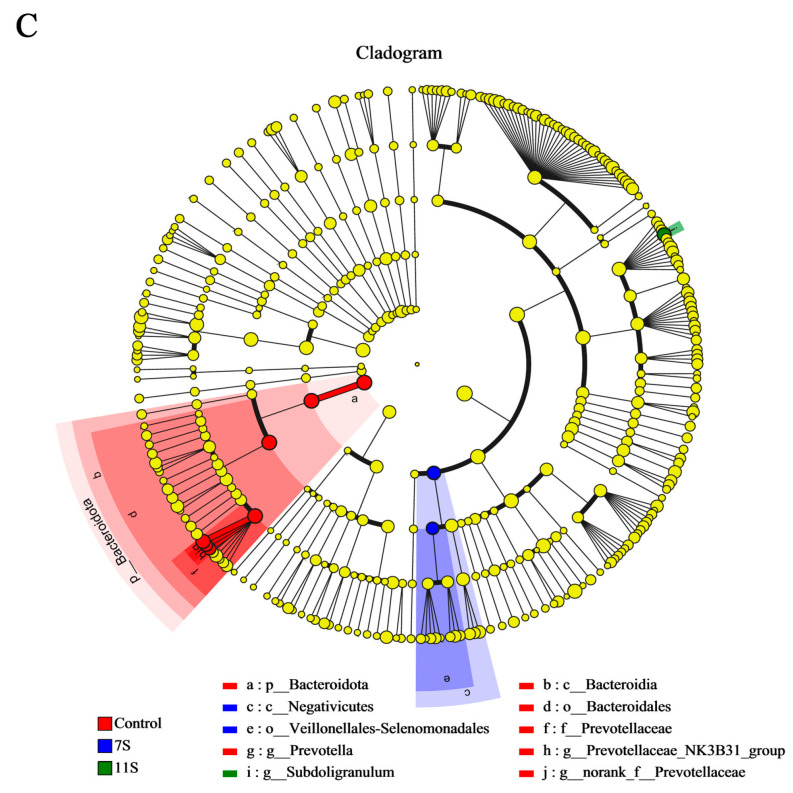
(**A**) The ratio of Firmicutes and Bacteroidetes. (**B**) LEfSe multi-level species difference discriminant analysis. (**C**) Evolutionary branch diagram. Data are shown as mean ± standard deviation (SD). ** *p* < 0.01, compared with the control group.

Interestingly, it was shown that 11S soybean globulin reduced the diversity and abundance of intestinal flora in weaned piglets, whereas 7S glycinin induced different results (as shown in Table 3). The reason for this result may be the cationic nature and hydrophobic basic structure of 11S glycinin, which shows comparable or even stronger anti-pathogenic and spoilage effects than penicillin, while the structure of 7S glycinin is not cationic in nature [17]. However, both 7S and 11S glycinin increased the relative abundance of pathogenic bacterial genera (*Corynebacterium* and *Micrococcus*) and decreased the relative intestinal abundance of beneficial bacterial genera (*Lactobacillus* and *Prevotella*). At the phylum level, 7S and 11S glycinin significantly increased the Firmicutes/Bacteroidetes ratio (Figure 4A), which is a marker of intestinal flora disorders and is negatively correlated with the degree of intestinal health [18]. In addition, as shown in Figure 4B,C, forty-seven species differed significantly among the three groups, with 7S and 11S glycinin altering the relative abundance of the most dominant colonies (LDA > 4). Compared with the control group, the relative abundance of *Bacteroidota* and *Prevotellaceae* in allergic groups (7S group and 11S group) decreased, while the relative abundance of *Firmicutes*, *Veillonellales-Selenomonadales* and *Micrococcus* rariorum increased. Therefore, these results indicated that 7S and 11S glycinin can induce intestinal flora disorder in piglets, and dysbacteriosis was also one of the direct causes of diarrhea.

### 2.3. 7S/11S Induced Intestinal ER Stress in Piglets 

**Figure 5:**
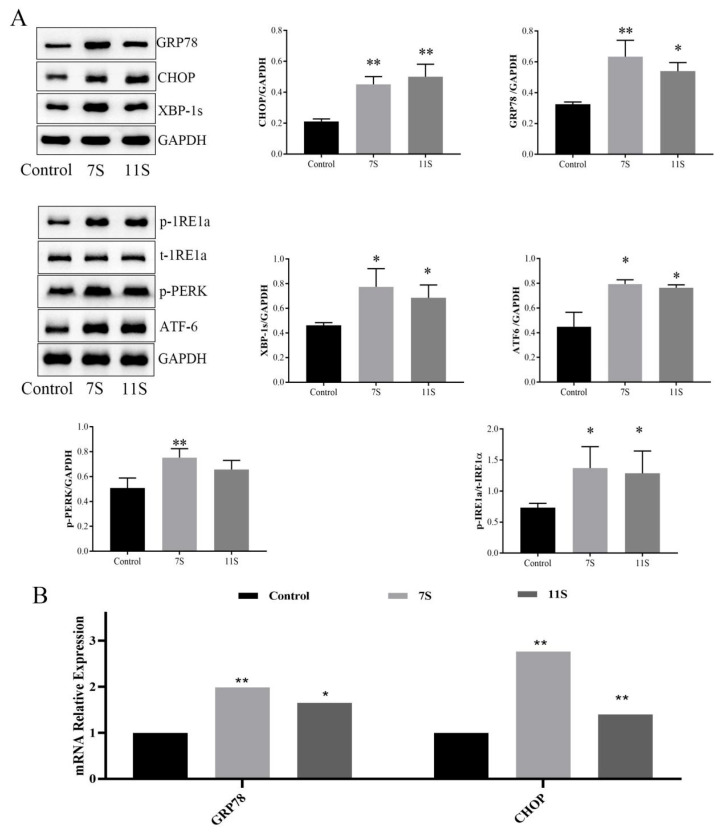

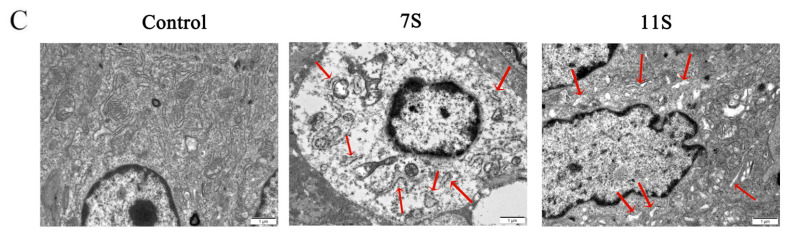
(**A**) The protein expression of Grp78, CHOP, p-IRE1α, XBP-1s, p-PERK and ATF-6 were measured using Western blot analysis. (**B**) mRNA levels of Grp78 and CHOP were measured using qRT-PCR analysis. (**C**) The ultrastructure of intestinal epithelial cells in piglets was observed using a transmission electron microscope; scale bar = 1 μm. The red arrow indicates the damaged endoplasmic reticulum in IPEC-J2 cells. Data are shown as mean ± standard deviation (SD). * *p* < 0.05, ** *p* < 0.01, compared with the control group.

Disrupted gut flora makes the gut more susceptible to stress-related damage [19]. Meanwhile, many studies have suggested that over-activation of mast cells during allergic reactions and intestinal mucosal barrier dysfunction are associated with ER stress [20]. Under ER stress, Grp78 is separated from three ER transmembrane sensors (IRE1, PERK, and ATF6) that are activated, which triggers adaptive UPR to restore protein homeostasis initially and promote the adaptation of cells to ER stress [21]. If the attempt to recover from ER stress fails, the unresolved ER stress will change the UPR from an adaptive to an apoptosis-promoting reaction mediated by CHOP and induce apoptosis to remove stress cells [1]. Analysis of ER homeostasis in the intestine revealed that 7S and 11S caused sharp increases in the mRNA and protein expression of Grp78 and CHOP (*p* < 0.05; Figure 5A,B). Meanwhile, marked elevations in the phosphorylation of IRE1α and PERK, the protein levels of XBP-1s and ATF-6 (*p* < 0.05 or *p* < 0.01; Figure 5B), along with ER dilatation and breakdown of the lamellar structure, were observed in 7S and 11S glycinin groups (Figure 5C). These findings demonstrate that 7S/11S-induced growth performance decline and ER stress in piglets.

### 2.4. 7S/11S Impaired Autophagic Flux in Weaned Piglets

**Figure 6:**
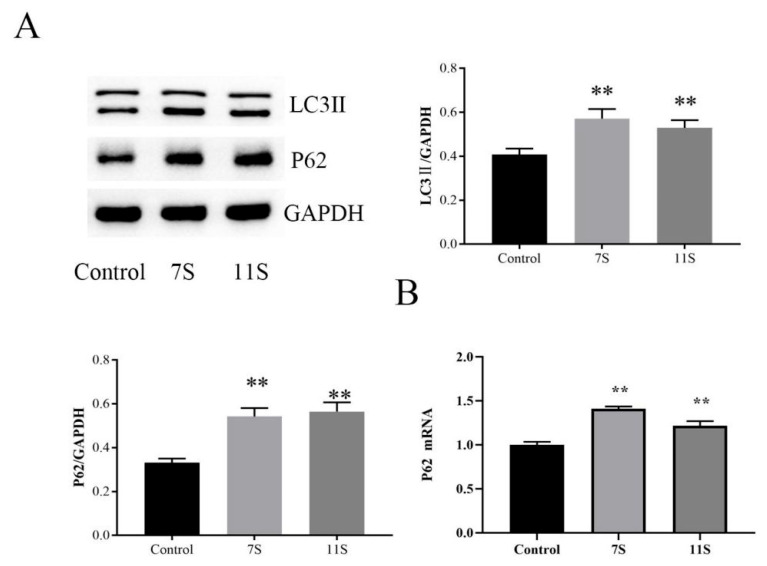
(**A**) The protein levels of LC3II and p62 were measured using Western blot analysis. (**B**) The relative expression levels of *p62* mRNA were measured using qRT-PCR analysis. Data are shown as mean ± standard deviation (SD). ** *p* < 0.01, compared with the control group.

Autophagy is an effector mechanism of ER stress as it achieves the metabolic needs of the cell by removing misfolded protein aggregates and damaged ER [22]. Studies have shown that autophagy is involved in the immunopathology of inflammatory diseases [23]. p62 protein is an autophagy receptor that directly binds to the autophagy regulator LC3, promoting the degradation of ubiquitinated protein aggregates, and is considered an important indicator for evaluating autophagy flux [24,25]. p62 protein expression increases when autophagy is inhibited and decreases when autophagy is activated. In this study, 7S and 11S upregulated the expression level of LC3II and p62 in the jejunum (*p* < 0.01; Figure 6A). The mRNA level of *p62* in the 7S group increased significantly (*p* < 0.01; Figure 6B) compared with control group. These data suggested that 7S and 11S blocked autophagic flux in the intestinal. Overall, blockage of autophagic flux increased the accumulation of unfolded and/or misfolded proteins in the ER, exacerbating 7S- and 11S-induced ER stress in weaned piglets. 

### 2.5. 7S/11S Induced Intestinal Barrier Damage in Piglets 

**Figure 7:**
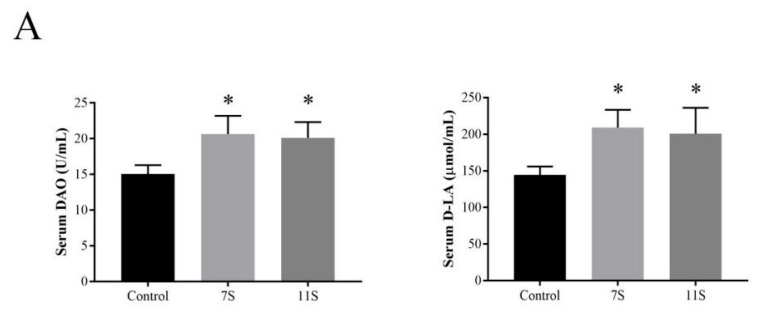

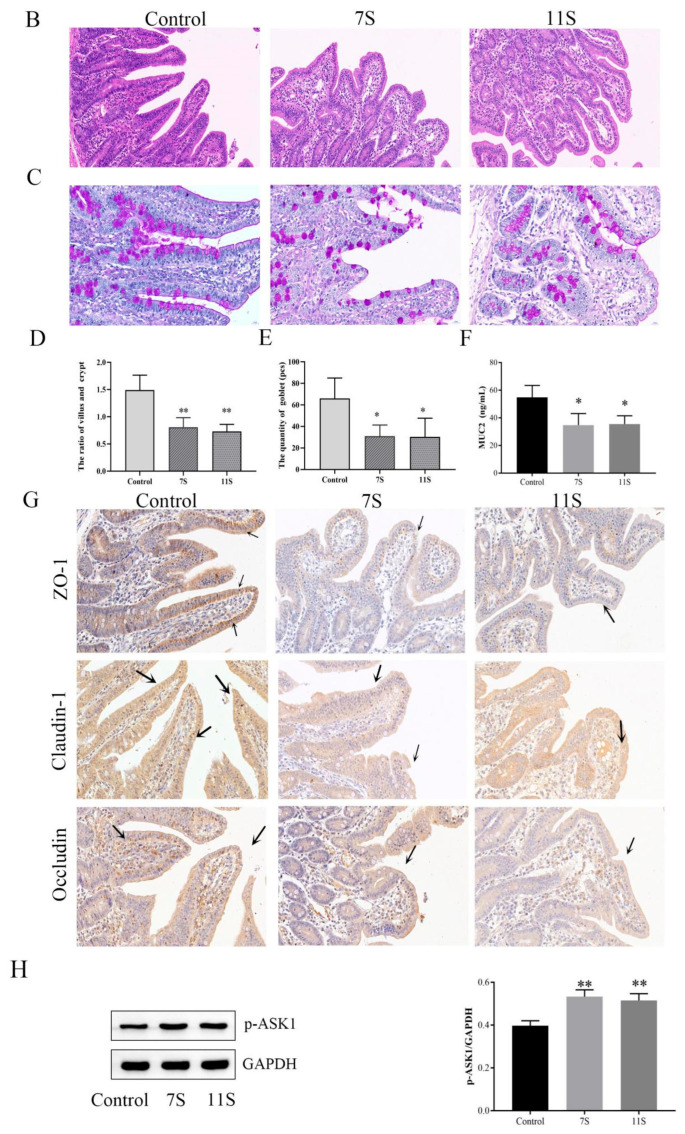

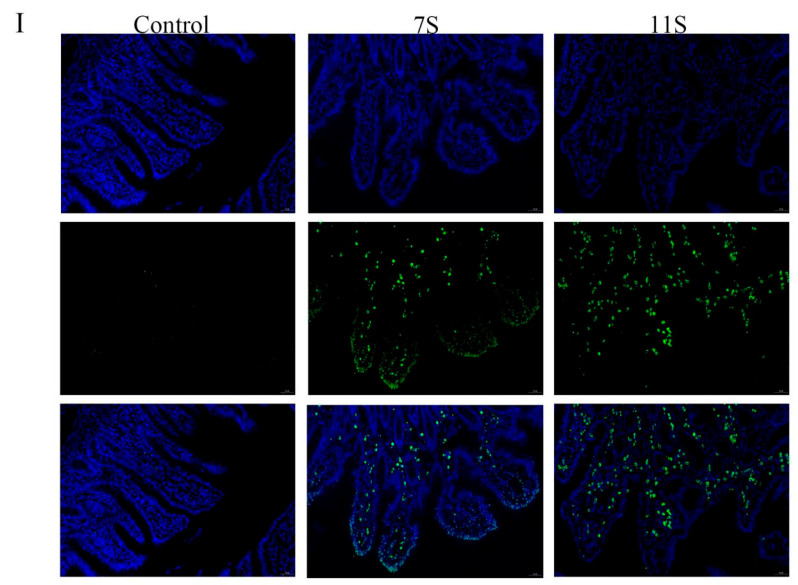
(**A**) The serum levels of DAO and D-lactate were measured using ELISA kits. (**B**) HE staining was used to assess intestinal villi and crypts (400×). (**C**) The GCs were observed using PAS staining (400×). (**D**) The ratio of villi and crypts. (**E**) The quantity of GCs. (**F**) The content of MUC2 was measured using ELISA kits. (**G**) The positive expressions of tight junction proteins (ZO-1, claudin-1, and occludin) were detected by IHC. Black arrows indicate localization and expression of target proteins. (**H**) The expression levels of p-ASK1 were measured by Western blot in the jejunum of piglets. (**I**) The intensity of fluorescence expression of TUNEL staining was statistically analyzed. Green fluorescence indicates apoptotic cells. Blue fluorescence labeled nuclei, green fluorescence labeled apoptotic cells. Data are shown as mean ± standard deviation (SD). * *p* < 0.05, ** *p* < 0.01, compared with the control group.

We detected the contents of serum DAO and D-LA in piglets, which reflect the integrity of and the degree of damage to the intestinal barrier [26,27]. The results showed that the levels of serum DAO and D-LA in the 7S and 11S groups were significantly higher than those in the control group (*p* < 0.05; Figure 7A). Compared with control group, histopathological examination of jejunal tissues indicated 7S- and 11S-induced apparent mucosal destruction, manifesting as denuded villi, decreased goblet cells and decreased villus height to crypt depth ratio (*p* < 0.05; Figure 7B−E).

In addition, 7S and 11S reduced the expression levels of recombinant Mucin 2 (Muc2) and tight junction proteins (ZO-1, claudin-1, and occludin) but increased protein expression of recombinant apoptosis signal-regulating kinase 1 (ASK1) in the jejunum (*p* < 0.05; Figure 7F−H). Under stress conditions, PERK-mediated translation inhibition is beneficial for weakening protein folding in the ER, but it also reduces TJ expression and hinders mucin synthesis in goblet cells [28], which damages the integrity of the intestinal barrier and allows pathogenic factors (bacteria and toxins) to enter the lamina propria, eventually leading to inflammatory reactions and intestinal injury [5]. Furthermore, IRE1α activates c-Jun N-terminal kinase (JNK), which then forms a tripartite complex with apoptosis signal-regulated kinase 1 (ASK1) and tumor necrosis factor (TNF) receptor-associated factor 2 (TRAF2) to induce the expression of inflammatory genes [29,30]. We detected apoptosis using the TUNEL assay, where green fluorescence represents apoptotic cells (Figure 7I). Therefore, this may be the reason why soybean antigenic protein induced overexpression of p-ASK1 and epithelial cell apoptosis (Figure 7I) in this experiment, as well as intestinal inflammatory damage in our previous studies [14]. These results further highlight the key role of ER stress in causing intestinal mucosal barrier damage [22].

## 3. Materials and Methods

### 3.1. Reagents and Antibodies

The 7S glycinin and 11S glycinin were provided by Professor Shuntang Guo of China Agricultural University (patent number: 200 410 029 589.4, Beijing, China). Primary antibodies against phosphorylated IRE1α (p-IRE1α; #ab48187), glucose-regulated protein 78 (GRP78; #ab108615), recombinant apoptosis signal-regulating kinase 1 (ASK1, #ab278547), recombinant activating transcription factor 6 (ATF6; #ab37149), and glyceraldehyde phosphate dehydrogenase (GAPDH; #ab9485) were obtained from Abcam (Cambridge, UK). Primary antibodies against C/EBP-homologous protein (CHOP; #mAb2895), LC3 (#mAb12741), sequestosome-1 (p62; #mAb39749), and XBP-1s (#mAb40435) were purchased from Absin Bioscience Inc. (Shanghai, China). Primary antibodies against phospho-protein kinase R-like ER kinase T982 (p-PERK; #AP0886), enzyme-linked immunosorbent assay (ELISA) kits for histamine (HIS), immunoglobulin E (IgE), 5-hydroxytryptamine (5-HT), IL-18, IL-1β, D-lactic acid (D-LA), diamine oxidase (DAO), and secreted mucin-2 (MUC-2) were purchased from Shanghai Youxuan Biotechnology Co., Ltd. (Shanghai, China). The PrimeScript™ RT Reagent (TRIzol) kit was obtained from Takara (Beijing, China).

### 3.2. Animal Treatment and Sample Collection

All animal care and experimental protocols were approved by the Ethics Committee of the Anhui Agricultural University (approval number 20210512). Thirty healthy weaning piglets (Duroc × [Landrace × Yorkshire], 21 d of age, 7.34 ± 1.30 kg) obtained from Han Shiwei Food Co., Ltd. (Bengbu, China) were randomly assigned to three experimental groups: control group (fed basic diet), 7S glycinin group (fed basic diet + 6% 7S), and 11S group (fed basic diet + 6% 11S). The nutritional formula of the basic diet is shown in Table 4. The supplementation ratio of 7S and 11S glycinin was based on our previous study. Each experimental group consisted of 10 piglets per pen.

Piglets were fed twice a day with free access to water. Feed intake and body weight of piglets were measured daily to calculate the average daily gain (ADG), average daily feed intake (ADFI), and feed-to-weight ratio (F/G). The ambient temperature in the pigsty was 26–28 °C and the humidity was 50–60%. The trial period was seven days. At 27 days of age, five piglets in each group were randomly selected and sacrificed via intracardiac injection of sodium pentobarbital (50 mg/kg body weight). Blood samples, intestinal tissues and cecum contents were collected for further examination.

### 3.3. Intestinal Permeability Assay

DAO and D-LA levels were determined using ELISA kits.

### 3.4. Histopathological Assay

Paraformaldehyde-fixed jejunum samples were embedded in paraffin wax, cut into three-micron sections, dewaxed in xylene, rehydrated, stained with hematoxylin and eosin, and analyzed using scanning electron microscopy (Pannoramic MIDI, 3D HISTECH, Budapest, Hungary). Jejunal goblet cells were stained with Alcian Blue/periodic acid–Schiff stain, as previously reported [31]. Three replicates of each group were randomly selected to calculate villus height, crypt depth, and cup cell density.

### 3.5. qRT-PCR Assay

RNA was extracted from piglet jejunum tissue samples and isolated using TRIzol reagent (TaKaRa, #RR047A, Kyoto, Japan), and the content, purity, and quality were determined using a UV spectrophotometer and electrophoresis. The samples were then stored at −80 °C. The primer sequences used for the qRT-PCR are listed in Table 5. qRT-PCR was performed as previously described [14].

### 3.6. Western Blot Analysis

Western blotting was performed as previously described [14]. The gray value of the protein bands was visualized using a ChemiDoc Imaging System (Bio-Rad, Hercules, CA, USA) and analyzed using ImageJ software (version number: V1.8.0).

### 3.7. Cecal Microbiome Composition Analysis

Fifteen piglets (five piglets per group) were randomly selected for 16S rRNA MiSeq sequencing. Cecal genomic DNA was extracted from 50 mg of cecal samples using the E.Z.N.A. Soil DNA Kit (Omega Bio-tek, Norcross, GA, USA) following the manufacturer’s protocol. The concentration and purity of extracted bacterial DNA were determined using a NanoDrop 2000 spectrophotometer (Thermo Scientific, Wilmington, DE, USA). The forward (5′-ACTCCTACGGGAGGCAGCAG-3′) and reverse (5′-GGACTACHVGGGTWTCTAAT-3′) primers were used to amplify the highly variable V3–V4 region of the 16S rRNA gene. PCR was performed using TransGen AP221-02: TransStart FastPfu DNA Polymerase (Illumina, San Diego, CA, USA), with a 20 µL reaction system, and an ABI GeneAmp Model 9700 PCR thermal cycler. 

DNA extraction, PCR amplification, and product purification and sequencing were performed with the assistance of Shanghai Meiji Biopharmaceutical Technology Co. Referring to the preliminary quantification results of electrophoresis, the PCR products were detected and quantified using the QuantiFluor™-ST Blue Fluorescence Quantification System (Promega, Beijing, China), and the TruSeqTM DNA Sample Prep Kit was selected for library construction. The purified amplicons were pooled at equimolar levels on the Illumina MiSeq PE300/NovaSeq PE250 platform (Illumina, San Diego, CA, USA) followed by paired-end sequencing.

### 3.8. Periodic Acid–Schiff (PAS) Staining

The fixed duodenum, jejunum, and ileum samples were dehydrated. After embedding in paraffin, the samples were cut into ultra-thin sections (3 microns), stained with Alcian blue for 10 min, and then rinsed thrice with distilled water for 5 min. The samples were oxidized in periodic acid solution for 15 min and then rinsed with running water. After immersion in Schiff stain solution in the dark, the samples were incubated in a 37 °C incubator for 20 min and rinsed with running water for 10 min. Hematoxylin staining and salt wine differentiation were then performed. Tap water was used to reverse the blue for 10 min. The samples were then rinsed slowly with distilled water for 3 min. After being dehydrated and sealed, they were observed and photographed using a scanning electron microscope (Pannoramic MIDI, 3D HISTECH, Budapest, Hungary).

### 3.9. Statistical Analysis

The experimental data are expressed as the mean ± standard deviation. LSD significance tests and one-way ANOVA were performed using SPSS 20.0 software. GraphPad Prism 7.0 was used for histogram plotting. The gut microbiota was selected for analysis on the Biosign Cloud platform provided by Shanghai Meiji Biotechnology Co., Ltd., Shanghai, China. The abundance of the gut microbiome was assessed by operational taxonomic unit (OTU), followed by alpha and beta diversity analysis, species clustering analysis, and linear discriminant analysis (LDA) effect size (LEfSe) analysis to determine the differences between the two groups of microbial communities. A 16S rRNA functional prediction of cecum microbes in weaned piglets was performed based on the Kyoto Encyclopedia of Genes and Genomes (KEGG) and Clusters of Orthologous Groups COG databases. *p* < 0.05 was considered as significant.

## 4. Conclusions

In summary, dietary supplementation of soybean antigen protein (7S glycinin and 11S glycinin) induced allergy and intestinal flora disorder in piglets. Meanwhile, it further triggered intestinal ER and blocked autophagic flux, which in turn damaged the intestinal barrier, leading to diarrhea and growth performance decline in weaned piglets. These findings suggest a new strategy for the prevention and treatment of allergic intestinal diseases induced by soybean antigen proteins. They also provide a theoretical basis for our upcoming verification of molecular mechanisms in vitro.

## Figures and Tables

**Table 1:** Effects of 7S and 11S on performance of weaned piglets.

Item	Control	7S	11S
Initial weight/kg	7.4 ± 0.86	7.15 ± 1.82	7.42 ± 1.46
Average daily gain (ADG, kg/d)	0.279 ^A^	0.15 ^B^	0.138 ^B^
Feed:Gain (F/G)	1.907 ^A^	3.46 ^B^	3.71 ^C^

Marked with different uppercase letters are highly significant differences (*p* < 0.01), and no letter or the same letter means the difference is not significant (*p* > 0.05).

**Table 2:** Effect of soybean 7S/11S globulin on intestinal flora α diversity in weaned piglets.

Items	Shannon Index	Simpson Index	Ace Index	Chao Index	Valid Sequences
Control	4.04 ± 0.24	0.04 ± 0.08 ^a^	480.75 ± 104.44	484.69 ± 115.85	38,019.80 ± 2743.40
7S	4.47 ± 0.37 ^a^	0.03 ± 0.01 ^a^	582.25 ± 92.97 ^a^	588.52 ± 87.44 ^a^	33,729.60 ± 3190.81
11S	3.50 ± 0.58 ^b^	0.08 ± 0.04 ^b^	433.02 ± 97.24 ^b^	425.05 ± 114.33 ^b^	36,424.40 ± 4874.57

Note: Data in the same column marked with different lowercase letters are significant (*p* < 0.05) and no letter or the same letter means the difference is not significant (*p* > 0.05).

**Table 3:** Abundance of dominant cecum bacteria at the phylum and genus level in weaned piglets.

Level	Classification	Control	7S	11S
Phylum	*Firmicutes*	56.77 ± 19.11	61.71 ± 9.53	74.99 ± 12.09
	*Bacteroidota*	36.99 ± 19.05 ^A^	26.99 ± 7.36 ^A^	9.08 ± 5.33 ^B^
	*Actinobacteriota*	1.87 ± 1.56 ^a^	2.77 ± 1.92 ^a^	14.50 ± 14.32 ^b^
Genus	*Lactobacillus*	18.58 ± 8.03 ^a^	8.61 ± 3.94	5.95 ± 2.17 ^b^
	*Prevotella*	20.07 ± 12.54 ^A^	9.95 ± 7.38	2.49 ± 1.67 ^B^
	*Collinsella*	1.56 ± 1.31 ^a^	2.21 ± 1.84 ^a^	11.91 ± 11.05 ^b^
	*Subdoligranulum*	1.82 ± 0.45 ^A^	2.58 ± 1.34 ^A^	10.91 ± 5.30 ^B^
	*Blautia*	5.42 ± 4.27	2.93 ± 2.97	3.65 ± 2.45
	*Faecalibacterium*	5.55 ± 4.95	2.29 ± 2.61	3.78 ± 2.80
	*Alloprevotella*	5.48 ± 6.20	3.36 ± 3.01	2.51 ± 3.80

Data in the same line marked with different lowercase letters are significant (*p* < 0.05), marked with different uppercase letters are highly significant (*p* < 0.01), and no letter or the same letter means the difference is not significant (*p* > 0.05).

**Table 4:** Composition and nutrient levels of basic diets.

Items	Content	
	Suckling (7–21 Day)	Weaned (21–28 Day)
Ingredients, %		
Corn	/	60.85
Dried whole milk	46.00	/
Skimmed milk powder	42.20	/
^a^ Soybean meal(expanded)	/	25.00
Whey powder	10.50	5.00
Fish meal	/	5.00
CaHPO_4_	/	2.20
Limestone	/	0.69
Wheat bran	/	0.37
Nacl	0.30	0.25
^b^ Premixb	1.00	0.49
Choline chloride	/	0.15
Total	100	100
Nutrient levels		
CP, %	27.9	20.80
Ca, %	1.04	0.64
TP, %	0.89	0.51
Lys, %	1.61	1.06
DE, MJ/kg	14.5	13.50

Note: ^a^ Expanded for the removal of antigenic proteins. ^b^ Per kg premixed feed (pre/post weaning):vitamin A, 7000/5250 U; vitamin D_3_, 2000/1050 U; vitamin E, 10/4.5 U; vita min K_3_, 2.2/1.2 mg; vitamin B_1_, 2.375/0.375 mg; vitamin B_2_, 4.8/1.8 mg; vitamin B_6_, 0.15/0.15 mg; vitamin B_12_, 17.5/7.5 mg; niacin, 16/6 mg; Ca-pantothenate, 5.75/3.75 mg; folic acid, 0.85/0.15 mg; biotin, 17.5/7.5 μg; lysine, 0.95/0.75 mg; antioxidant, 45/45 μg; enzyme preparations (amylase, protease, lipase, and phytase of mixture of amylase, protease, lipase and phytase), 1100/1000 mg; flavoring agent, 45/40 mg; sweetener, 45/40 mg; neomycin, 0/20 mg; Cu (as copper sulfate), 15/15 mg; Fe (as ferrous sulfate), 144/144 mg; Zn (as zinc sulfate), 110/110 mg; Mn (as manganese sulfate), 20.18/10.18 mg; I (as calcium iodide), 0.4/0.4 mg; Se (as sodium selenite), 0.35/0.3 mg.

**Table 5:** Primer sequences for qRT-PCR amplification.

Gene	Forward Primer (5′→3′)	Reverse Primer (5′→3′)	Product (bp)
*GRP78*	GGCTCTACTCGCATCCCAAAG	CCTGAACAGCAGCACCGTAA	115
*CHOP*	CTTCACCACTCTTGACCCTG	CACTTTGTTTCCGTTTCCTG	170
*p62*	CCCGCGTTCCCTACAAA	GGCTGAAACAGAAGCTGAAG	181
*GAPDH*	TGACCCCTTCATTGACCTCC	CCATTTGATGTTGGCGGGAT	160

## Data Availability

All relevant data are within the paper.
